# Apoptosis Induced by Ziziphora tenuior Essential Oil in Human Colorectal Cancer Cells

**DOI:** 10.1155/2021/5522964

**Published:** 2021-07-22

**Authors:** Mohammadreza Azimi, Jalil Mehrzad, Armita Ahmadi, Elnaz Ahmadi, Ali Ghorbani Ranjbary

**Affiliations:** ^1^Department of Biochemistry, Medical Faculty, Saveh Branch, Islamic Azad University, Saveh, Iran; ^2^Department of Microbiology and Immunology, Faculty of Veterinary Medicine, University of Tehran, Tehran, Iran; ^3^Research Institute of Agricultural, Ferdowsi University of Mashhad, Mashhad, Iran; ^4^Faculty of Chemistry, Islamic Azad University of Tehran, Iran

## Abstract

Ziziphora (Cacotti in Persian) belongs to the Lamiaceae family (mint group) and is vastly found in Iran and Asia. This traditional medicinal plant is normally used as analgesic and for treatment of particular gastrointestinal diseases. Since colorectal cancer is one of the most common causes of death in the world and the second leading cause of cancer death among adults, there is a pressing need to inhibit this malignancy by using methods with minimal side effects. One of these methods is the use of natural resources such as medical plants. This study is aimed at investigating the expression of apoptosis-related genes in the adjacent culture of colorectal cancer epithelial cells (HT-29) with Ziziphora essential oil (ZEO). The essential oil was extracted from Ziziphora leaves, and its compounds were determined and then added to the HT-29 culture medium at different concentrations. After 24 hours, the HT-29 cells were harvested from the medium and cytotoxicity was analyzed by MTT assay. After MTT assay and determination of the percentage of apoptosis by flow cytometry, RNA extraction was performed and the expression levels of Bax, Bcl-2, caspase 3 (C3), and caspase 9 (C9) were analyzed using newly designed primers by reverse transcription (RT) qPCR method and GeniX6 software. Also, specific antibodies were used for western blot analyses of those molecules. GC analysis revealed 42 different compounds in the ZEO, including pulegone (26.65%), menthone (5.74%), thymol (5.51%), and menthol (1.02%). MTT assay showed that the concentration of 200 *μ*g/ml of ZEO had the highest HT-29 cell death during 24 hours. After incubation with the concentration of 50 *μ*g/ml of ZEO for 24 and 48 hours, caspase 3 and 9 gene expressions in the treated group increased compared to those in the control group (*P* < 0.001), while the Bcl-2 expression decreased. The results showed that having anticancer compounds, ZEO can increase C3 and C9 and decrease Bcl-2 expressions, causing apoptosis in HT-29 cells *in vitro*. This can lead to the use of ZEO as a factor for colorectal cancer treatment.

## 1. Introduction

Cancer has always been one of the most fundamental issues of human health. Despite a large number of researches and developments in the past decade, cancer remains one of the most important causes of death globally. According to the most recent statistics, cancer is the second leading cause of death in the world after cardiovascular disease [1–3]. Recent studies have shown that colorectal cancer (CRC) is the second most common cancer in women after breast cancer and the most common cancer in men after lung cancer [4, 5]. CRC is one of the most common malignancies that cause many deaths annually and the second most deadly cancer worldwide with about 881,000 deaths in 2018 [6]. Nowadays, different methods including surgery, chemotherapy, and radiotherapy are used to treat cancer, but one of the side effects of these methods is the loss of healthy cells, which has led researchers to move towards new methods of treatment by reducing side effects [6, 7]. Using medical plants and their extracts as a treatment for cancer has attracted attention, and a lot of research on this area has recently been conducted. HT-29, adenocarcinoma cell, is one of the most widely used epithelial-derived cell lines to mimic the behavior of epithelial cell cancer and CRC therapy in laboratory and clinics [8, 9].

Ziziphora is a traditional medicinal plant in the family Lamiaceae. This plant is a subshrub, its height is between 20 and 50 cm, and the leaves are small, opposite, almost lanceolate, and without petioles. It also has small and complete flowers in white, pink, and purple. Its medicinal properties can be used in the treatment of digestive disorders such as diarrhea and colic [10]. Besides, Ziziphora has antibacterial [11, 12], antioxidant [13], and intestinal disinfectant [12] effects. Ziziphora extract has also been shown to increase the activity of caspases 3 and 9 through the internal pathway of apoptosis, causing apoptosis and necrosis even in larvae of hydatid cyst [14, 15]. Further, the alcoholic extract of the aerial part of Ziziphora, due to its compounds such as menthol, can cause cytotoxicity in various cancerous cells (e.g., HT-29 and T-47D cell lines).

The most important Ziziphora phytochemical compounds are thus the anticancer ones including pulegone, menthol, and menthone. In recent studies, the anticancer effects of these compounds have been observed. Its mechanism is through the effect on various messaging pathways such as apoptosis, cell viability, and proliferation [14–16]. Ziziphora has effective substances such as cineole, piperitone, menthone, pulegone, isomenthol, and curcumin [11–16].

Therefore, according to the results of previous studies [14, 15] and the mechanism of the unknown effect of Ziziphora essential oil (ZEO) on HT-29 cells in the present study, our current study is aimed at investigating the expression of apoptotic-related genes in the adjacent culture of colorectal epithelial cancerous cells with ZEO.

## 2. Materials and Methods

### 2.1. Ziziphora Essential Oil (ZEO) Preparation Method

The leaves and branches of Ziziphora tenuior were dried in shade and ground by a grinding machine. Then, the essential oil was extracted by water distillation in a Clevenger apparatus (type apparatus 1928) for 3 hours. The ratio of essential oil to the dry weight of the plant was 5%. The harvested ZEO was then stored in dark glass for subsequent use.

First, ZEO was extracted and then its compounds were identified by a GC-MS (HP*-*6840/5973) spectrometer in the central laboratory of Ferdowsi University of Mashhad. The components were identified by comparing their mass spectrum with the existing standard spectrum.

The rest of the present study was performed in the central laboratory of Faculty of Veterinary Medicine, University of Tehran, and the biotechnology department of Ferdowsi University of Mashhad in 2019 with a code of ethics IR.MUMS.REC.1398.42456. The HT-29 cell line was purchased from the biotechnology department (cell bank). Cells were cultured inside a flask with filter cap containing 90 ccs RPMI 1640 medium (Gibco, USA) enriched with 10% fetal bovine serum (FBS) and 100 *μ*l antibiotics (penicillin 0.01 *μ*g/*μ*l and streptomycin 0.01 *μ*g/*μ*l) in an incubator at 37°C and carbon dioxide 5%. Then, the cells were passaged, separated, according to the desired seeding density calculations, and added to 3 cm and/or 6-well culture plates after counting.

### 2.2. MTT Assay

MTT is one of the water-soluble yellow tetrazolium salts, reduced by dehydrogenases in cell-activated mitochondria, and then deposits as insoluble formazan crystals in living cells. These crystals are purple, and their purple color value is proportional to cell activity and the number of living cells. Briefly, 100 *μ*l/ml of medium containing cells at a density of 5 × 10^4^ cells/ml was seeded in each well of a flat-bottom 96-well plate. Cells were permitted to adhere to the plate for 24 h (5% CO_2_ and 37°C). Then, 100 *μ*l RPMI 1640 medium containing different concentrations of (0, 50, 100, and 150 *μ*g/ml) ZEO was incubated for 24 h. After the above time, 20 *μ*l MTT with a concentration of 5 g/l was added to each well and placed in the incubator at 37°C for 3 hours. DMSO (100 *μ*l/ml) was used as the positive control, and wells were left with no cells for the negative control. Finally, after 15 minutes of incubation at room temperature, the optical absorption of the plate was measured by a spectrophotometer at 570 nm. To obtain better results and ensure the accuracy of the obtained results, each experiment was repeated five times. The percentage of the viable cells was calculated using the following formula: 100 × mean treatment absorbance/mean control absorbance; likewise, calculate percentage cytotoxicity with the following equation, using corrected absorbance: %cytotoxicity = [100 × (control − sample)].

### 2.3. Cell Culture and Grouping

After initial culture and counting, 3 × 10^5^ HT-29 cells/ml were transferred to 6-well plates and divided into two groups including group 1, HT-29 cells, according to the results of MTT, with 50 *μ*g/ml ZEO, and group 2, only HT-29 cells as the control group. After 24 and 48 hours of culture, mRNA and protein level expressions are evaluated by RT-PCR and western blot, respectively.

### 2.4. Primer Design and Quality Analysis by Agarose Gel

In this study, after obtaining exon sequences from NCBI (National Center for Biotechnology Information) and Ensembl, investigated primers were designed on two exons or as forward or reverse on the junction of two exons by Beacon Designer. Then, using Beacon, Oligo, and NCBI, Primer-BLAST was performed and primers were investigated for the position and extra bands. Then, after ordering and purchasing primers, they were diluted and used according to the manufacturer's protocol (Table 1).

Total RNA extraction was performed using Dena Zist Asia (S-1010-1), Iran. After analyzing nanodrop and agarose gel, it was converted to cDNA by Yekta Tajhiz Azma (YTA (Cost No. YT4500, Iran)) kit. Then, for denaturation, according to the binding temperature, cDNA strands were heated at 95°C for 10 minutes; then, a 40-cycle period consisting of 95°C in 10 seconds, 60°C in 20 seconds, and 72°C in 20 seconds was used for PCR, and the quality analysis of primer-based PCR products was investigated primers in 2% agarose gel.

### 2.5. Flow Cytometry

To check apoptosis in the HT-29 colon cancer cells treated by the extract of Ziziphora tenuior L., the ZEO-treated and untreated groups of HT-29 cells were assessed by flow cytometry (BD FACSAria III) using annexin V and PI according to the appropriate instruction. The HT-29 cells were treated with ZEO (50 *μ*g/ml) and 0 *μ*g of ZEO/ml (as untreated/control group cells) for 24 and 48 hours. Finally, the percentage of various stages of apoptotic HT-29 cells was reported.

### 2.6. RT-qPCR and Analysis

All biological samples were placed in Rotor-Gene Q 2.3.5 RT-PCR, and 95°C cDNA denaturation temperature was performed for 10 minutes; then, a 40-cycle period including 95°C in 10 seconds, 60°C in 20 seconds, and 72°C in 20 seconds was used for Bax, Bcl-2, caspase 3, and caspase 9 and reading. In this experiment, Yekta Tajhiz Azma (YTZ (Cost No. YT2551, Iran)) Master Mix and SYBR Green kits were used. At first, crew standard was performed for normalization of cDNA. Then, the melting curve and CT were analyzed in each sample.

The initial analysis was performed by the GeneX v6.7, and the results were calculated in delta, delta CT, and log-2. Then, statistical analysis was performed using GraphPad Prism 8.

### 2.7. Western Blotting

To determine protein expression, western blot analysis was performed. Briefly, after 24 h of treatment with ZEO, the HT-29 cells were lysed with 70 *μ*l of PhosphoSafe™; then, protein concentration (20 *μ*g) was calculated by BCA protein analysis. Electrophoresis was performed using Nu-PAGE 10% SDS-PAGE Bis-Tris gel in SDS-PAGE buffer. Polyvinylidene fluoride membrane (PVDF) was used for transfer. Next, membrane was blocked with bovine serum albumin (3%). Afterwards, membranes were washed with Tris-buffered saline containing Tween 20 (TBST) and incubated overnight with primary antibody (procaspase-3 (catalog no. sc-7148; anti-rabbit), procaspase-8 (catalog no. sc-7890; anti-rabbit), procaspase-9 (catalog no. sc-7885; anti-rabbit), Bcl-2 (catalog no. sc-492; anti-rabbit), BAX (catalog no. sc-493; anti-rabbit), and *β*-actin (catalog no. sc-47,778; anti-mouse)) diluted 1 : 1000. After that, membrane was washed three times with TBST and secondary antibody (1 : 1000) was added to be incubated for 1 h and washed with TBST. Then, band intensities were detected using a chemiluminescent substrate SuperSignal Femto kit and band densities were analyzed using ImageJ 1.52a program (Bethesda, Maryland, USA) [16].

### 2.8. Statistical Analyses

All the experiments were performed in duplicate, being the results expressed as mean ± SEM of three independent experiments. The collected data were analyzed using *t*-test. All analyses were carried out using GraphPad Prism 8.

## 3. Results

**Figure 1:**
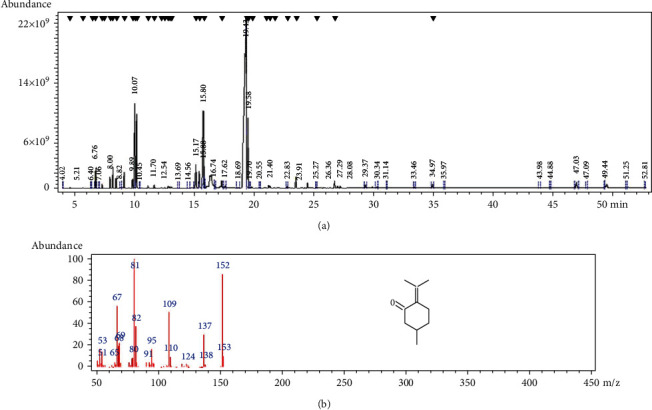
The existence of pulegone in ZEO GC/MS. (a) Chromatogram of Ziziphora essential oil (ZEO). (b) Chemical structure of pulegone in ZEO.

The main compounds of Ziziphora essential oil measured by GC/MS are shown in Table 2.  Figure 1 shows the Ziziphora essential oil chromatogram. 42 chemical compounds were identified. Among these, 7 compounds make up the most ZEO chemicals, given in order as follows: pulegone (26.65%), alpha-terpinyl acetate (9.53%), geraniol (7.11%), menthone (5.74%), thymol (5.51%), alpha terpineol (3.24%), and menthol (1.05%) were present in respective decreasing order (Figure 1).

### 3.1. MTT Assay Revealed Proapoptotic Properties of ZEO on Epithelial Cancer Cells

**Figure 2:**
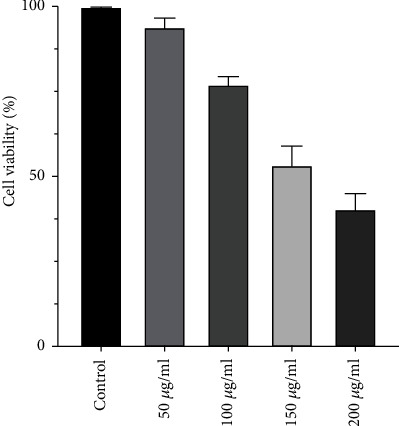
Percentage of the viability of treated HT-29 cells with different concentrations of Ziziphora essential oil (ZEO).

This assay was performed to identify the concentration of IC_50_. The highest death was obtained in the concentration of 200 *μ*g/ml at 24 hours after treatment (Figure 2). Then, according to the MTT results, the concentration of 50 *μ*g/ml of ZEO was used to analyze its effect on the expression of apoptosis-associated genes. Indeed, according to the optical density-based MTT assay of ZEO un/treated HT-29 cells, the ZEO behaved in a dose-dependent manner in these cancerous cells.

### 3.2. Flow Cytometry Results Confirmed ZEO's Proapoptotic Activity on Epithelial Cancer Cells

**Figure 3:**
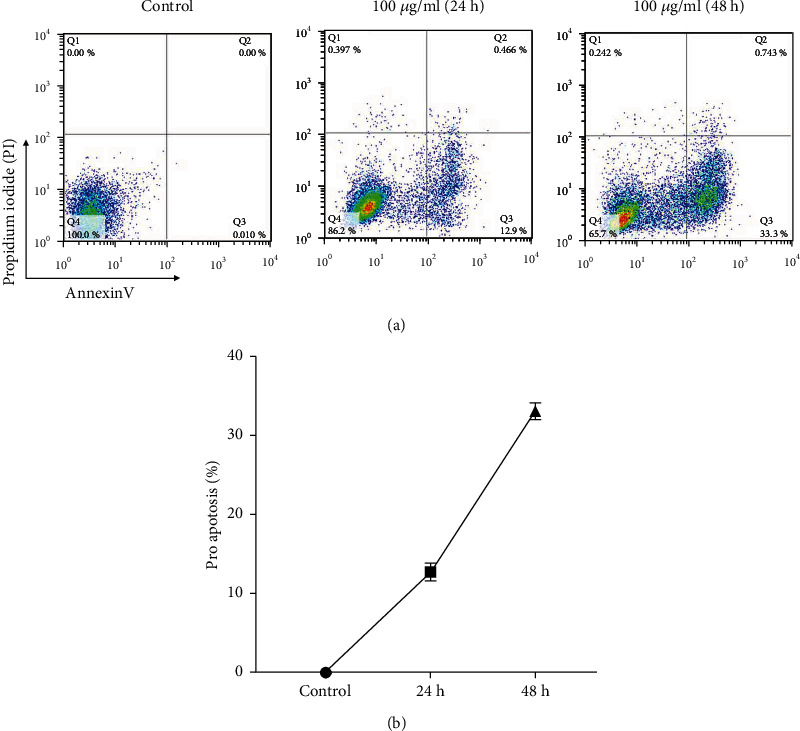
Effect of concentration of 50 *μ*g/ml of Ziziphora essential oil (ZEO) on apoptosis in HT-29 cells. (a) Flow cytometry figure (annexin v/PI), Q4 healthy cells, Q3 early apoptosis, Q2 late apoptosis, and Q1 necrosis. (b) Percentage of early apoptotic cells after 24- and 48-hour incubation of HT-29 cells with ZEO.

**Figure 4:**
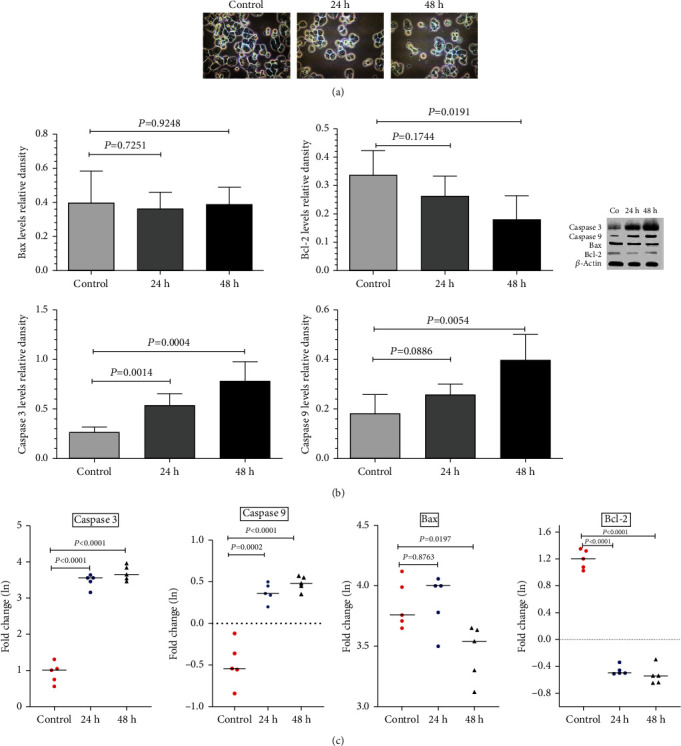
Ziziphora essential oil- (ZEO-) induced apoptosis includes an increase in caspase 3 and 9 and a decrease in Bcl-2 and Bax expressions in HT-29 cells. (a) Light microscopic pictures (×100 magnification) with arrows represent the morphological changes caused by apoptosis in the examined HT-29 cells. (b) Bax, Bcl-2, C3, and C9 expression results obtained by western blot in HT-29 cells. (c) mRNA, Bax, Bcl-2, C3, and C9 results obtained by RT-PCR.

Flow cytometry on HT-29 cells was performed to pinpoint how ZEO affected cell death (apoptosis or necrosis). Data analysis was performed using software following the division of a two-dimensional annexin V versus PI curve into four regions/quadrants (Qs) (Q1, Q2, Q3, and Q4). In this division, Q1 represents necrotic HT-29 cells with annexin V^−^ and PI^+^; the Q2 region represents the late stage of apoptotic HT-29 cells with characteristics of annexin V^+^ and PI^+^; the Q3 region represents healthy cells with annexin V^−^ and PI^−^; and the Q4 region represents early apoptotic HT-29 cells with characteristics of annexin V^+^ and PI^−^. The average of Q1, Q2, Q3, and Q4 in ZEO-challenged HT-29 cells after 48 h was 0.26, 0.70%, 34.33%, and 64.62%, respectively (Figure 3). The 24 and 48 hours of incubation of HT-29 cells with 50 *μ*g/ml of ZEO caused time-dependent apoptosis (Figure 3). The percentage of early apoptotic HT-29 cells was 12.35 ± 1.96 and 33.54 ± 2.12, respectively (Figure 3(b)). Microscopic results also confirmed the results so that the number of cells in the treated groups with Ziziphora tenuior L. after 24- and 48-hour incubation was lower than that of untreated (control) ones (Figure 4(a)). Also, changes in cell morphology and apoptosis following ZEO challenge were evident.

### 3.3. Altering the Levels of Bax, Bcl-2, C3, and C9 Gene Expression in ZEO-Exposed Epithelial Cancer Cells

Caspase 3 and 9 expressions at the protein level in the Ziziphora tenuior L.-treated groups (24 and 48 hours) increased compared to those in the control group, but Bax expression did not show a significant change. The Bcl-2 expression decreased only in the 48-hour treated group with Ziziphora tenuior L. compared to the control group (*P* = 0.0191) (Figure 4(b)).

Figure 4(c) shows the expression of the studied genes at the mRNA level; the results showed that caspase 3 and 9 expressions at the mRNA level in the treated groups with Ziziphora tenuior L. (24 and 48 hours) increased compared to those in the control group (*P* < 0.0001), but the expression level of Bcl-2 in the treated groups decreased significantly compared to the control group (*P* < 0.0001). Bax expression only in the Ziziphora tenuior L. group treated for 48 hours decreased significantly compared to that in the control group (*P* = 0.0197). Similar results on the protein levels were finally confirmed with western blotting of the protein expression of Bax, Bcl-2, C3, and C9.

## 4. Discussion

The results of this study showed that the most abundant compound of Ziziphora essential oil is pulegone. Although a comparison of the chromatography results of ZEO (Figure 1 and Table 2) with the extracts of oils of other Ziziphora species shows partial similarities among them, but still some dissimilarities on its chemical composition might mainly be due to the growing conditions of the plant, water and air conditions, place of growth, altitude, etc. (20–23). Various studies have shown that pulegone is the main ingredient in various Ziziphora species (20–25); nonetheless, the percentage of pulegone in the present study is lower than that in other studies.

Also, the concentration of 50 *μ*g/ml of ZEO reduces the number and imposes morphological changes in HT-29 cells. Additionally, the expression of caspases 3 and 9 in ZEO-treated cells with increasing ZEO showed an increase in their expressions compared to the control group. These changes can lead cells to apoptosis. Different studies have shown pulegone can stimulate apoptosis [17, 18]. Pulegone causes apoptosis by reducing NF-*κ*B activity [19]. Also, the investigated ZEO contained menthol and menthone that can cause apoptosis [20]. In 2013, the toxic effect of four medicinal plants including Ziziphora clinopodioides Lam. on epithelial cells of colorectal cancer was investigated and the results showed that Ziziphora clinopodioides Lam. has antitumor properties [21]. Another study in 2016 examined the chemical compounds and anticancer effects of aerial parts of Ziziphora clinopodioides Lam. The toxic effect of this plant on cell lines of colorectal cancer (HT-29), breast cancer (T-47D), leukemia (K-562), and mouse embryonic fibroblasts was investigated. The results indicated an extraordinary inhibitory and toxic effect of Ziziphora clinopodioides Lam. compounds on these cancerous cell lines that are consistent with the present study.

**Figure 5:**
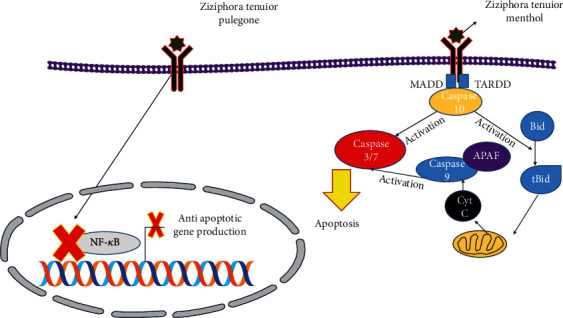
Schematic view of the effect of Ziziphora essential oil (ZEO) on apoptosis through pulegone and menthol. On the other hand, menthol activates caspase 10, followed by activation of the Bid pathway, and caspases 3 and 7 cause apoptosis.

In this study, the main extracted compounds were pulegone (24%), menthol (14%), and menthone (9%) [22]. Other studies showed a tumor suppressor mechanism so that P53 acts as a transcription factor for a set of proapoptotic proteins from the BCL family (Puma, Bid, Noxa, and Bax). It eventually induces mitochondrial permeability and releases cytochrome *c*. Cytochrome *c* is essential for apaf1 activation; this protein is vital in activating the caspase activation pathway. P53 also induces ASC (apoptosis-associated speck-like protein), which plays a role in the positioning of Bax protein in the mitochondria and induction of mitochondrial membrane permeability for cytochrome *c* release [23–25]. In Figure 5, the mechanism of apoptosis by Ziziphora pulegone and menthol is presented. Various studies have shown that pulegone reduces NF-*κ*B followed by apoptosis [26–28]. Roy et al. showed that pulegone reduces inflammation caused by LPS by reducing the effects of NF-*κ*B [29]. Souldouzi et al. also showed that pulegone causes apoptosis in mouse ovarian follicular cells [28]. Menthol is another important compound of ZEO. Various studies have shown that menthol activates caspases 3 and 7 through caspase 10 affecting HSP90, followed by apoptosis [30–32].

The results of the present study showed that ZEO increases the levels of caspases 3 and 9 at mRNA and protein levels in HT-29 cells and decreases the amount of Bcl-2. It seems highly likely that the compounds such as menthol and polygon could cause apoptosis in HT-29 cells through the NF-*κ*B pathway as well as activate caspases through the TRPM8 channel [23–25, 33]. Chemical analyses of ZEO showed that among the 42 various compounds in analyzed ZEO, pulegone (26.65%), alpha-terpinyl acetate (9.53%), and geraniol (7.11%) were the main compounds, which might have broad effects on cancer cells.

Indeed, here we used only cell lines and only a single concentration of ZEO, which is inadequate to draw a strong conclusion; as such, examining the effects of various concentrations of ZEO on a particular normal cell line is warranted. Nonetheless, the results showed that ZEO due to having anticancer compounds such as menthol and pulegone can increase the expression of C3 and C9 and decrease Bcl-2, causing apoptosis in HT-29 cells *in vitro*. These can be courageous points to the application of ZEO as a medicinal plant of choice for the treatment of CRC.

## Figures and Tables

**Table 1:** List of different RT-qPCR primers used in the study.

Gene (ENST)	Sequence (5′–3′)	*T*_m_ (°C)	Length (bp)
Caspase 3 (ENST00000393585.6)	F: ATGGGAGCAAGTCAGTGGAC	60	84
	R: CGTACCAGAGCGAGATGACA		
Caspase 9 (ENST00000469637.1)	F: GGCGGAGCTCATGATGTCTGTG	61	156
	R: TTCCGGTGTGCCATCTCCATCA		
Caspase 8 (ENST00000391871.4)	F: GGATGGCCACTGTGAATAACTG	60	101
	R: TCGAGGACATCGCTCTCTCA		
Bcl-2 (ENSG00000126453)	F: GAGCGTCAACAGGGAGA	60	164
	R: GCCAGGAGAAATCAAACA		
Bax (ENSG00000087088)	F: ACTAAAGTGCCCGAGCTGA	60	161
	R: ACTCCAGCCACAAAGATGGT		
*β*-Actin (ENST00000515712.1)	F: CTACCTTCAACTCCATCA	60	165
	R: GAGCAATGATCTTGATCTTC		

**Table 2:** Composition of Ziziphora tenuior essential oil from Iran.

	Components	^∗^RI	Chemical formula	^∗∗^RT	^∗∗∗^LRI	Identification	%
1	Pulegone	1214	C_10_H_16_O	18.11	1570	RI, MS	26.651
2	*α*-Terpinyl	960	C_12_H_20_O_2_	14.45	970	RI, MS	9.533
3	Geraniol	1211	C_10_H_18_O	14.80	1274	RI, MS	7.114
4	Menthone	985	C_10_H_18_O	13.91	990	RI, MS	5.744
5	Thymol	2010	C_10_H_14_O	21.50	2020	RI, MS	5.512
6	*α*-Terpineol	1011	C_10_H_18_O	15.56	1097	RI, MS	3.247
7	Menthol	2035	C_10_H_20_O	22.35	2065	RI, MS	1.051
8	Octanol	983	C_8_H_18_O	11.43	989	RI, MS	0.965
9	*α*-Pinene	930	C_10_H_16_	6.76	939	RI, MS	0.865
10	Camphene	943	C_10_H_16_	7.29	950	RI, MS	0.861
11	Sabinene	964	C_10_H_16_	29.96	972	RI, MS	0.88
12	*β*-Pinene	979	C_10_H_16_	8.20	976	RI, MS	0.758
13	Myrcene	871	C_10_H_16_	14.62	991	RI, MS, ^1^H-NMR	0.965
14	Limonene	1010	C_10_H_16_	10.07	1020	RI, MS, ^13^C NMR	1.035
15	*α*-Terpinene	995	C_10_H_16_	11.86	1018	RI, MS	0.51
16	Eucalyptol	1020	C_10_H_18_O	10.26	1069	RI, MS	0.125
17	*γ*-Terpinene	1050	C_10_H_16_	11.16	1060	RI, MS	0.1258
18	Linalool	1080	C_10_H_18_O	12.53	1097	RI, MS	0.115
19	Terpinolene	1075	C_10_H_16_	19.29	1089	RI, MS	0.356
20	Isomenthone	1141	C_10_H_18_O	15.47	1148	RI, MS, ^13^C NMR	0.458
21	Isomenthol	1102	C_10_H_20_O	15.23	1115	RI, MS, ^13^C NMR	0.18
22	Piperitenone	1340	C_10_H_16_O	31.14	1346	RI, MS	0.256
23	Carvacrol	1296	C_10_H_14_O	30.57	1303	RI, MS	0.198
24	Epi alpha cadinol	1210	C_15_H_26_O	35.41	1218	RI, MS	0.056
25	Spathulenol	1567	C_15_H_24_O	42.10	1585	RI, MS, ^1^H-NMR	0.145
26	Nerolidol	1573	C_15_H_26_O	18.41	1582	RI, MS, ^1^H-NMR	0.11
27	*δ*-Cadinene	1440	C_15_H_24_	17.69	1446	RI, MS, ^1^H-NMR	0.35
28	*γ*-Cadinene	1531	C_15_H_24_	38.60	1535	RI, MS	0.44
29	*β*-Bisabolene	1507	C_15_H_24_	8.24	1514	RI, MS	0.49
30	Germacrene-D	1474	C_15_H_24_	37.28	1481	RI, MS, ^1^H-NMR	0.756
31	Eugenol	1380	C_10_H_12_O_2_	24.52	1384	RI, MS	0.668
32	Eucalyptol	1030	C_10_H_18_O	10.22	1032	RI, MS	0.12
33	2-Nonen-1-ol	758	C_9_H_18_O	11.93	771	RI, MS	0.189
34	cis-*β*-Farnesene	894	C_15_H_24_	28.20	923	RI, MS	0.106
35	*γ*-Elemene	1102	C_15_H_24_	29.69	1437	RI, MS, ^1^H-NMR	0.1
36	Carvone	1223	C_10_H_14_O	31.50	1240	RI, MS	0.08
37	Cyclohexanone	1254	C_6_H_10_O	19.41	1260	RI, MS	0.09
38	Butanoic acid	1205	C_4_H_8_O_2_	22.36	1209	RI, MS	0.05
39	*β*-Bourbonene	1385	C_15_H_24_	21.54	1392	RI, MS	0.1
40	Caryophyllene	1415	C_15_H_24_	34.61	1417	RI, MS, ^1^H-NMR	0.03
41	Humulene	1454	C_15_H_24_	25.86	1455	RI, MS, ^1^H-NMR	0.07
42	4,7-Dimethoxy-5-[prop-1-en-1-yl]-2H-1,3-benzodioxole	900	C_12_H_14_O_4_	34.99	912	RI, MS, ^1^H-NMR	0.056

^∗^RI: retention indices calculated on apolar; ^∗∗^RT: retention time (min); ^∗∗∗^LRI: retention indices of literature.

## Data Availability

The datasets generated during and/or analyzed during the current study are available from the corresponding author on reasonable request.
